# Ultrasound versus electromyography for the detection of fasciculation in amyotrophic lateral sclerosis: systematic review and meta-analysis

**DOI:** 10.1590/0100-3984.2019.0055

**Published:** 2020

**Authors:** Márcio Luís Duarte, Wagner Iared, Acary Souza Bulle Oliveira, Lucas Ribeiro dos Santos, Maria Stella Peccin

**Affiliations:** 1 Escola Paulista de Medicina da Universidade Federal de São Paulo (EPM-Unifesp), São Paulo, SP, Brazil.; 2 Centro Universitário Lusíada (Unilus) - Fundação Lusíada, São Paulo, SP, Brazil.

**Keywords:** Ultrasonography, Electromyography, Fasciculation, Amyotrophic lateral sclerosis, Ultrassonografia, Eletroneuromiografia, Fasciculação, Esclerose lateral amiotrófica

## Abstract

The objective of this study was to determine the diagnostic accuracy of ultrasound and electromyography for the detection of fasciculation in patients with amyotrophic lateral sclerosis and to compare detection rates between the two methods. By searching the Cochrane Library, MEDLINE, Excerpta Medica, and Latin-American and Caribbean Health Sciences Literature databases, we identified studies evaluating the diagnostic accuracy and fasciculation detection rates of ultrasound and electromyography. The Quality Assessment of Diagnostic Accuracy Studies, version 2, and RTI item bank tools were used for the evaluation of methodological quality. Ultrasound, for 10 s or 30 s, had a higher detection rate than did electromyography in all muscles evaluated. The overall detection rate (in patients) did not differ significantly between ultrasound for 10 s and ultrasound for 30 s. The accuracy of ultrasound for 10 s was 70% in muscles and 85% in patients. The accuracy of ultrasound for 30 s was 82% in patients. Ultrasound provided detection rates superior to those achieved with electromyography, independent of the examination time and muscles evaluated.

## Abstract (pt)

O objetivo deste estudo foi determinar a acurácia diagnóstica da ultrassonografia e da eletroneuromiografia para o diagnóstico da fasciculação e comparar suas taxas de detecção. Foram realizadas buscas nas bases de dados eletrônicas Cochrane Library, MEDLINE, Embase e Lilacs, para estudos que avaliam a acurácia diagnóstica e as taxas de detecção da ultrassonografia e eletroneuromiografia. As ferramentas Quality Assessment of Diagnostic Accuracy Studies, versão 2, e RTI item bank foram utilizadas para avaliação da qualidade do método. A ultrassonografia, tanto de 10 s quanto de 30 s, apresentou taxa de detecção superior à eletroneuromiografia em todos os músculos avaliados. A avaliação da taxa de detecção por pacientes não apresentou diferença significativa entre a ultrassonografia de 10 s e 30 s. A acurácia da ultrassonografia de 10 s nos músculos foi de 70%, enquanto nos pacientes foi de 85%. Já na ultrassonografia de 30 s, a acurácia nos pacientes foi de 82%. A ultrassonografia apresentou taxas de detecção superiores à eletroneuromiografia, independentemente do tempo de sua avaliação e dos músculos avaliados.

## INTRODUCTION

Fasciculations are rapid, random, fine, flickering, or vermicular twitching movements of a group of muscle fibers innervated by a single motor unit^(1-7)^. Although fasciculation is almost obligatory among patients with amyotrophic lateral sclerosis (ALS), which is the most common motor neuron disease and the one with the highest mortality, it can also occur in other diseases and conditions^(1,2,4-6,8-12)^:

- lower motor neuron (LMN) diseases, including syringomyelia, Creutzfeldt-Jakob disease, radiculopathy, spinal muscle atrophy, multifocal motor neuropathy, and peripheral neuropathy (acquired and inflammatory)
- metabolic disorders, including hyperparathyroidism, hyperthyroidism, and hypomagnesemia
- conditions induced by drugs such as caffeine, lithium, terbutaline, anti-cholinesterase, and theophylline
- after exercise, stress, or anxiety, as well as spontaneously, in healthy individuals

Fasciculation is a clinical and electromyographic marker of ALS, particularly when it is generalized and is accompanied by muscle loss or electromyographic changes indicative of denervation^(13,14)^.

Fasciculation may be detected by clinical evaluation, electromyography (EMG), or ultrasound^(15-17)^. As diagnostic methods, EMG and ultrasound each have advantages and disadvantages. However, there is as yet no screening protocol for either method.

Currently, EMG is the gold standard for assessing LMN function in ALS^(1,18)^. For the diagnosis of LMN disease, fasciculation potentials should be detected in several regions^(1)^. The sensitivity of EMG is dependent on the duration of screening of each muscle and the number of muscles evaluated^(15,19)^.

Ultrasound is highly sensitive to movement, allowing good visualization of fasciculation^(1,19,20)^. Minimal movements as small as 5 µm are detectable, and the temporal resolution (frame rate) is more than 80 fps^(1)^. Neither computed tomography nor magnetic resonance imaging has that advantage, because they are static exams^(18)^.

Studies have shown that EMG assesses only the superficial musculature, is limited in terms of the area it can study, is not capable of evaluating atrophy, and takes 10-90 s to detect a fasciculation^(1,21-23)^. In contrast, ultrasound assesses the superficial and deep musculature, thereby allowing a greater number of motor units to be studied^(24,25)^, is able to identify atrophy, as well as to calculate the cross-sectional area of evaluation, and takes only 8-10 s to detect a fasciculation^(1,21,22)^.

Ultrasound is a noninvasive, painless method that is more widely available than is EMG, as well as being less expensive and not involving the use of radiation^(1,12,14,26-31)^. However, ultrasound does not differentiate between benign (stable) and malignant (unstable) fasciculations. That differentiation is made by using EMG to assess motor unit potentials^(22,32)^. There is currently no one diagnostic modality capable of detailing all events occurring in a muscle or muscle group over time^(1)^.

The objectives of this study were to determine the accuracy of ultrasound and EMG for the detection of fasciculation, to compare the rate of fasciculation detection using ultrasound and EMG in patients with ALS, to determine which muscles are better assessed with ultrasound and EMG, and to evaluate the ability of ultrasound to identify muscle atrophy.

## METHOD

This was a systematic review of studies of diagnostic accuracy, as defined in the Cochrane Handbook for Systematic Reviews of Diagnostic Test Accuracy, version 5.1. Studies evaluating the diagnostic accuracy of ultrasound and EMG for the detection of fasciculation were included regardless of publication status. There was no language restriction.

The study was approved by the local institutional review board, and the review was previously registered with the International Prospective Register of Systematic Reviews (Registration no. CRD42017078388).

From reference journals, we selected relevant articles or abstracts that were deemed potentially eligible for inclusion. Two authors, working independently, identified the eligible texts. In cases of disagreement, a third author was consulted. Data were extracted through the use of a standardized form.

Eligible studies with a control group were evaluated using a quality assessment tool, the Quality Assessment of Diagnostic Accuracy Studies, version 2^(33)^. In all eligible studies, including those with a control group, we used the RTI item bank questionnaire, which is a tool focused on the evaluation of biases and precision^(34,35)^.

We searched the Cochrane Library, MEDLINE, Excerpta Medica, and Latin-American and Caribbean Health Sciences Literature databases for studies published up through January of 2019. We also evaluated the bibliographies of the included studies and the main review articles on the subject. Manual searches of the bibliographies were also conducted. For all analyses and diagrams, we employed the software Review Manager, version 5.3 (RevMan 5; Cochrane Collaboration, Oxford, UK) and Meta-DiSc, version 1.4 (Cochrane Colloquium, Barcelona, Spain).

### Studies selected

We identified 139 studies on the subject and selected 12 that met the inclusion criteria. Two of those studies were excluded: one because ultrasound and EMG were performed at the same time, so the study was not blinded^(36)^; another because it did not contain all the necessary data^(18)^. Therefore, ten studies were included in the review and meta-analysis^(15,21,26,37-43)^. Of those ten studies, six included a control group, allowing assessment of accuracy. The four studies that did not include a control group were included only in the analysis of the detection rate for each method.

Among the ten studies included, the duration of the ultrasound examination of each muscle was 10 s in four studies and 30 s in another four, whereas one study did not report the duration and both durations were employed in one study. For the last study, we separated the data in our analysis of the detection rates by ultrasound regardless of examination time, so that the patients who underwent the exam at both durations were not counted twice. We utilized the 30-s data for those patients, because all of the muscles that presented fasciculation within 10 s also presented fasciculation within 30 s. Data from the study that did not mention the duration of the ultrasound examination were used only in the analysis of the detection rates. In five studies, each muscle was evaluated individually, allowing us to evaluate the specific detection rate for each muscle.

**Figure 1:**
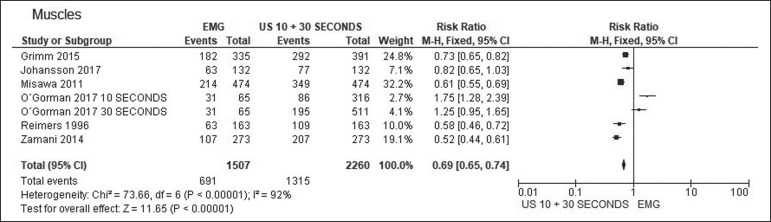
Forest plot. Comparison of EMG and ultrasound (10-s and 30-s examinations) in muscles.

**Figure 2:**
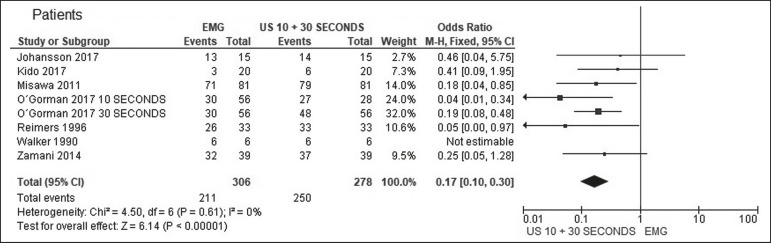
Forest plot. Comparison of EMG and ultrasound (10-s and 30-s examinations) in patients.

In all muscles, the rate of fasciculation detection was higher for ultrasound than for EMG, independent of the duration of the ultrasound examination. In the analysis of the detection rates in patients, that difference was even greater. For individual muscles and for patients, Tables 1, 2, and 3 compare the two methods at ultrasound examination times of 10 s, 30 s, and both, respectively. The forest plots in Figures 1 and 2 separate those data for muscles and for patients, respectively.

**Table 1:** Comparison between EMG and 10-s ultrasound examination in terms of the fasciculation detection rates in muscles and patients.

		EMG			10-s ultrasound examination	
Aspect	Number of studies	Muscles with fasciculation/muscles evaluated	Detection rate		Muscles with fasciculation/muscles evaluated	Detection rate
Muscle(s)						
Sternocleidomastoid	2	4/33	12.10%		27/102	26.40%
Tongue	2	47/85	55.42%		88/130	67.69%
All	4	339/695	48.77%		627/1262	49.68%
Patients	3	69/104	66.34%		96/104	92.30%

**Table 2:** Comparison between EMG and 30-s ultrasound examination in terms of the fasciculation detection rates in muscles and patients.

Aspect	Number of studies	EMG			30-s ultrasound examination	
		Muscles with fasciculation/muscles evaluated	Detection rate		Muscles with fasciculation/muscles evaluated	Detection rate
Muscle(s)						
Tongue	3	36/153	11.21%*		106/168	63.09%
Biceps brachii	2	73/120	60.83%		105/120	87.50%
Dorsal interosseous	2	69/120	57.50%		94/120	78.33%
Vastus lateralis	2	54/120	45.00%		85/120	70.83%
Tibialis anterior	2	56/120	46.66%		98/120	81.66%
All	3	352/812	43.34%		718/1226	58.56%
Patients	3	107/143	74.82%		133/143	93.00%

* One study did not detect fasciculation by EMG in any of the 81 patients evaluated, because there was continuous voluntary contraction or activity associated with respiratory motion.

**Table 3:** Comparison between EMG and ultrasound (10-s or 30-s examination) in terms of the fasciculation detection rates in muscles and patients.

		EMG			10-s or 30-s ultrasound examination	
Aspect	Number of studies	Muscles with fasciculation/muscles evaluated	Detection rate		Muscles with fasciculation/muscles evaluated	Detection rate
All muscles	6	691/1507	45.85%		1315/2260	58.18%
Patients	7	211/306	68.95%		250/278	89.92%

### Accuracy assessment

Two studies had a control group that described the number of muscles, thus allowing us to evaluate accuracy. Both studies used ultrasound examination times of 10 s. Five studies had a control group and reported the number of patients, thus allowing us to assess 10-s ultrasound accuracy. Two studies had a control group and reported the number of muscles, thus allowing us to evaluate accuracy. Both studies used ultrasound examination times of 30 s.

In two randomized clinical trials^(38,40)^, the 10-s ultrasound examination showed a sensitivity of 76% and a specificity of 73% for the detection of fasciculations in ALS, with a 95% confidence interval *p* < 0.05 and an accuracy of 70%. In five other randomized clinical trials^(15,37,38,40,41)^, the 10-s ultrasound examination showed a sensitivity of 75% and a specificity of 93% for the detection of fasciculations in ALS, with a 95% confidence interval *p* < 0.05 and an accuracy of 85%. In two other randomized clinical trials^(40,43)^, the 30-s ultrasound examination showed a sensitivity of 94% and a specificity of 80% for the detection of fasciculations in ALS, with a 95% confidence interval *p* < 0.05 and an accuracy of 82%. Therefore, in all examinations, the accuracy was higher than 70%.

## DISCUSSION

We found ultrasound to be the method with the best accuracy for detection of fasciculation, as previously reported in the medical literature^(26,36)^. The method is accurate for evaluating patients and for evaluating individual muscles, regardless of the duration of the examination. The difference between the 10- and 30-s ultrasound examinations for the detection of fasciculation was not significant, although the 30-s examinations provided more data for each muscle, allowing the analysis of greater numbers of patients and muscles. Ultrasound had a sensitivity and specificity higher than 70% for both evaluations (individual muscles and patients). The specificity was higher than 90% in the patients evaluated by ultrasound.

The muscles for which ultrasound presented the best detection rate (%) were the biceps brachii (88%) and the tibialis anterior (82%), both during 30-s examinations, followed by the vastus lateralis (71%), also during 30-s examinations. During the 10-s examinations, the detection rate was highest (68%) for the tongue muscle and lowest (26%) for the sternocleidomastoid muscle. The best detection rates achieved with EMG were for the biceps brachii muscles (61%) and the dorsal interosseous muscles (58%).

The technique of detecting fasciculation by ultrasound is easily learned, as evidenced by the level of interobserver agreement reported-100% for the presence or absence of fasciculations^(20,29,36,43,44)^. Ultrasound identifies fasciculations in 80% of cases, compared with only 45% for intramuscular EMG^(1)^. That could be explained by the fact that EMG is able to evaluate only a few motor units, many fewer than those evaluated by ultrasound^(41)^.

Fasciculations in specific muscle groups, such as those of the arm and trunk, as well as in the sartorius and tibialis anterior muscles, are of much greater diagnostic significance than are those detected in the quadriceps, calves, or hamstrings^(41)^. Fasciculations are more common in the distal muscles of the leg than elsewhere in the body^(42)^. It should be borne in mind that EMG evaluation of the tongue is quite limited because it is difficult to achieve complete relaxation of the tongue muscle^(45-47)^, as confirmed by Misawa et al.^(21)^, who were unable to detect fasciculation by EMG in the tongues of 81 patients evaluated, suggesting that ultrasound is more feasible for that task^(21)^.

Ultrasound and EMG both have disadvantages. There is as yet no single modality that provides all of the details of events that occur within a muscle or muscle group over a given period of time^(1)^. Ultrasound is a practical, painless technique that can allow earlier diagnosis and provide greater confidence in the diagnosis of ALS, due to the visualization of fasciculations^(21)^. Ultrasound is also valuable in making the differential diagnosis with similar diseases^(22)^.

Musculoskeletal ultrasound may add knowledge to neurophysiological test data by detecting changes in muscle morphology and echotexture that can imply denervation^(30,31)^. Ultrasound can also be utilized as a screening tool before submitting patients to procedures that are more invasive and painful, such as EMG and muscle biopsy^(48,49)^.

The advantage of ultrasound over EMG is its greater sensitivity in detecting fasciculations^(37)^. According to Grimm et al.^(37)^, the disadvantage of ultrasound is its inability to distinguish between stable and unstable fasciculations, which may be additional criteria for the diagnosis of motor neuron diseases. Currently, the distinction between stable and unstable fasciculations can be made only with EMG. In another study, Grimm et al.^(49)^ suggested that ultrasound is also able to detect muscle fasciculation in the early stages of sepsis. Extensive involvement of the arm muscles over time may be a sign of sepsis-induced muscle disorders^(49)^. That fact, together with the greater accessibility of ultrasound and its accuracy in the detection of fasciculation, may point to a new direction in its indications for uses other than the evaluation of LMN diseases, especially ALS, in which ultrasound may soon replace EMG as the best diagnostic method.

Given the high sensitivity of ultrasound for the detection of fasciculation, the method can contribute to the evaluation of this sign in other diseases, including metabolic diseases (hyperparathyroidism, hyperthyroidism, and hypomagnesemia) and conditions induced by the use of drugs (anticholinesterases, caffeine, lithium, theophylline, and terbutaline). Ultrasound has all of the necessary characteristics to be used as an ALS screening method, including low cost, availability, accessibility, high sensitivity, and high specificity, as well as being painless^(43)^. According to our findings in this review, ultrasound is suggested as a screening method for the evaluation of fasciculation in all motor neuron diseases, including ALS, thereby improving the rate of fasciculation detection and significantly reducing diagnostic costs.

In this systematic review, we have identified the need for new prospective clinical trials to evaluate muscles not yet studied as well as those for which there are no definitive results, such as the rectus femoris muscle, which has been evaluated in only one clinical trial^(37)^. The muscles for which the detection rates in the 30-s ultrasound examinations were highest were the biceps brachii and the tibialis anterior muscles.

Ultrasound can detect muscle atrophy, fatty infiltration, and intramuscular fibrosis^(49)^. However, in our searches, we identified no studies evaluating the use of ultrasound for the detection of atrophy in ALS.

## CONCLUSION

The importance of this systematic review is that we have shown ultrasound to be a more accurate method for the detection of fasciculation than is EMG. In comparison with EMG, ultrasound is more affordable and accessible, as well as being noninvasive, being able to evaluate more muscle fibers, and providing information on muscle atrophy.
